# Editorial: Wellbeing in parents of neurodivergent children

**DOI:** 10.3389/fpsyt.2024.1480313

**Published:** 2024-10-09

**Authors:** Lai-Sang Iao

**Affiliations:** Department of Psychology, Nottingham Trent University, Nottingham, United Kingdom

**Keywords:** wellbeing, mental health, parents, autism, neurodiversity

Parents of neurodivergent children often experience high levels of stress and report difficulties in accessing support (e.g., 1, 2). Multiple factors have been identified in understanding parenting stress and mental health in parents of neurodivergent children (e.g., 3, 4). This Research Topic presents original findings highlighting how various factors contributed to wellbeing in parents of neurodivergent children, shedding light on new approaches and interventions as well as informing practice and policy. These contributing factors can be categorised into child factors including problem behaviours (Chua et al.), sleep disturbances (Gálvez-Ortega et al.), and mental health (Piro-Gambetti et al.), parental factors including education level, occupation and working time (Zhu et al.), and negative attentional bias (Lovell et al.), intra-familial factors including parent-couple relationship and family income (Piro-Gambetti et al.; Zhu et al.), and extra-familial factors including social support for parents and intervention and services for autistic children and adults (Németh et al.; Xu et al..; Zhu et al.). The seven articles published in this Research Topic has provided new evidence and enriched the existing literature of wellbeing in parents of neurodivergent children, covering not only various contributing factors but also different populations from various countries across Eastern and Western cultures, including China, Hungary, Malaysia, United Kingdom and United States.

Child behavioural issues, including externalising and internalising behaviours such as hyperactivity and sleep problems, have been frequently examined in relation to parents’ wellbeing. However, different cultures may perceive and address these behaviours and parents’ wellbeing differently. Chua et al. reported a cross-sectional survey examining the relationship between behavioural issues in autistic children and caregiver burden in Sarawak, Malaysia where there was a diverse culture of Malays, Ibans, Chinese, and Bidayuh. Consistent with previous findings (e.g., 4, 5), there was a significant positive association between child behavioural issues, particularly hyperactivity/noncompliance, and caregiver burden. Piro-Gambetti et al. further indicated that child externalising mental health issues were longitudinally associated with depression symptoms in American fathers of autistic children 12 months later and this association was mediated by a parental factor, i.e., parent-couple relationship satisfaction. Together with other findings, they suggested that the longitudinal relationships among child mental health, parent depression and parent-couple relationship satisfaction were bidirectional and complex in families of autistic children. Gálvez-Ortega et al., on the other hand, drew data from a longitudinal caregiver-report study and revealed that reduced child sleep was associated with exacerbated caregiver stress and depression but not anxiety in caregivers of young children with and without neurogenetic syndromes which include Angelman, Prader Willi, Williams, and Fragile X syndromes.

Although depression is prevalent among parents of neurodivergent, especially autistic, children (e.g., 6, 7), British parents of autistic children did not seem to be characterised by an attentional bias toward the processing of negative information (Lovell et al.). Moreover, the higher the social support perceived the less excessive thinking about negative information (i.e., rumination) was reported by Chinese parents of autistic children and this association was moderated by the extent of intervention level received by their autistic children (Xu et al.). That is, high intervention level received by autistic children buffered the negative effect of low perceived social support on rumination in Chinese parents of autistic children. However, Németh et al.‘s qualitative study suggested that support and services for autistic adults were highly limited than those for autistic children in Hungary where parents of autistic adults perceived extreme difficulty in accessing support and services for their adult child, deteriorating their overall wellbeing. Unfortunately, this is not specific to Hungary but generalised across the globe (e.g., 8, 9). Extra-familial factors such as social support, intervention and services interacted with parental factors such as education level, affecting parenting efficacy in Chinese fathers of autistic children (Zhu et al.). They also provided insights into multiple pathways incorporating multiple parental, intra-familial and extra-familial factors to achieve parenting efficacy in Chinese fathers of autistic children.

In conclusion, the collective findings of these seven studies underscore the complexity of how various factors, which could be broadly categorised into child factors, parental factors, intra-familial factors and extra-familial factors, contributed to wellbeing in parents of neurodivergent children from five different countries across Eastern and Western cultures. Understanding the interactive effects of multiple factors on wellbeing in parents of neurodivergent children sheds light on the development of personalised support programs based on individual child and parent characteristics as well as intra-familial and extra-familial situations to effectively support each family of neurodivergent children. System-level interventions such as strategies to ensure the availability and quality of education and services for families of neurodivergent children, prioritising wellbeing in parents of neurodivergent children, are also highly beneficial.
